# Biomechanical and tissue reaction: the effects of varying sutures size on canine abdominal wall stitching

**DOI:** 10.3389/fvets.2023.1254998

**Published:** 2023-11-10

**Authors:** Shuai Li, Yizhe Guo, Xingkai Zhao, Dong Lang, Zhenlei Zhou

**Affiliations:** College of Veterinary Medicine, Nanjing Agricultural University, Nanjing, China

**Keywords:** suture size, abdominal wall, biomechanical, inflammation, tissue reaction, canine

## Abstract

**Objective:**

Larger diameter sutures can provide sufficient tensile strength to surgical incisions but may exacerbate the inflammatory response caused by the amount of implanted foreign material. This experiment aims to investigate the differences in biomechanical stability and tissue reactivity after suturing canine midline abdominal incisions with different suture sizes.

**Method:**

Assessing the biomechanical differences between USP 2-0, 3-0, and 4-0 PGA sutures using uniaxial tensile testing on *ex vivo* canine midline skin and fascial muscle tissues using either a simple continuous or simple interrupted technique. mRNA and protein expression levels of inflammatory factors were measured through RT-PCR and ELISA. Tissue reactivity was evaluated using a semi-quantitative scoring system.

**Result:**

For strains below 30% in skin and below 50% in muscle, there were no significant differences among groups. The results of skin biomechanical testing showed that the USP 4-0 PGA suture group demonstrated significantly lower maximum tensile strength compared to the USP 2-0 PGA or USP 3-0 PGA suture groups. However, it remained capable of providing at least 56.3 N (1.03 MPa) tensile strength for canine skin incisions, matching the tensile strength requirements of general canine abdominal wall surgical incisions. In addition, there were no statistically significant differences observed in the maximum tensile strength among different size of sutures according to the data of biomechanical testing in muscle. Larger diameter sutures led to increased levels of inflammatory factors (IL-1β, IL-6, TNF-ɑ) and tissue reactivity. Simple interrupted sutures caused higher levels of inflammatory factors in muscular tissue compared to simple continuous sutures.

**Conclusion:**

USP 4-0 PGA sutures provide sufficient biomechanical stability for suturing canine abdominal skin and linea alba. Suture size significantly influences tissue reactivity after suturing, with smaller gauge sutures reducing early tissue inflammatory response. Thus, USP 4-0 PGA suture has more advantages to suturing canine abdominal surgical incisions.

## 1. Introduction

Although laparoscopic surgery has been increasingly applied in recent years, open surgery remains the primary choice in many complex surgeries requiring a wider field of vision in small animal clinic. The most common approach for open surgery involves a midline incision into the abdomen, which entails the cutting and suturing of the skin and linea alba. Poor postoperative wound healing is a common complication following surgical procedures, manifesting as incisional hernia, wound dehiscence, and hypertrophic scarring (1). These complications not only result in increased postoperative care and treatment expenses but also pose significant threats to the life and health of postoperative patients, regardless of whether it is an incisional hernia or wound dehiscence, which are associated with high morbidity and mortality rates (2, 3).

Suboptimal surgical wound healing is influenced by multiple factors, including suture technique (4), infection (5), inflammation (6), anemia (7), diabetes (8), and obesity (9). Tissue healing encompasses a series of interconnected biological events, such as clotting, inflammation, proliferation, and remodeling (10). Inflammation plays a pivotal role in the wound healing process, serving as a necessary component for hemostasis and recruitment of non-specific immune cells. It safeguards the body against pathogenic invasion and facilitates the removal of necrotic tissue (11). However, numerous studies have indicated that excessive inflammation can lead to tissue damage, resulting in delayed healing (12, 13) and scar formation (14, 15). Administration of anti-inflammatory agents has shown significant potential in reducing wound inflammation levels and promoting healing (16, 17). Studies by Dovi et al. (18) demonstrated that neutrophil depletion accelerates the healing of full-thickness dermal wounds. Moreover, PU-1 knockout mice (lacking macrophages and functional neutrophils) exhibited minimal scarring following wound healing (19), indicating that neutrophils and macrophages may not be indispensable for wound healing, and their absence might actually facilitate scarless healing. Notably, fetal tissue exhibits rapid scarless healing, potentially attributed to considerably lower levels of inflammation compared to adult tissue (20, 21). Additionally, tissues known for their reduced scar formation propensity, such as oral mucosa and cornea, display diminished accumulation of polymorphonuclear cells and expression of inflammatory mediators during the wound healing phase (22, 23). Therefore, attenuating the inflammatory response at the wound site, particularly preventing the occurrence of additional inflammation, plays a crucial role in averting suboptimal wound healing outcomes.

Suturing of surgical incisions is necessary, as the use of surgical threads provides adequate mechanical support, bringing the edges of the incision closer together. This, in turn, shortens the inflammatory phase and reduces scar formation during the healing process. However, there is a paradox: all suture materials are foreign materials that can elicit a local immune response in the surrounding tissues. Consequently, the additional inflammation caused by sutures may potentially prolong the inflammatory stage and healing time of the wound, leading to adverse outcomes such as wound dehiscence and hypertrophic scarring (24). Currently, there is a widespread focus on the tissue reactivity differences caused by suture materials, ranging from relatively inert polypropylene to highly inflammatory catgut (25). However, scant literature has addressed the impact of differences in suture diameter on the inflammatory response resulting from the volume of suture material implanted.

Surgeons typically select sutures based on their experience, but research indicates that in most cases, surgeons may overestimate the tension requirements of the tissues to be sutured, resulting in the use of larger-sized suture needles and threads (26, 27). Studies have shown that the clinical use of USP 2-0 sutures can provide sufficient stability, resulting in a 0% incidence of incisional hernia in 356 human cases of midline abdominal closure (28). When selecting suture needles and threads, the smallest size that achieves the intended purpose of suturing should be chosen to minimize tissue trauma and foreign materials implantation. However, smaller-sized sutures imply lower tensile strength, requiring surgeons to strike a balance between suture tension and foreign materials implantation (29). Further research is needed to investigate the optimal specifications of suture needles and threads for surgical practitioners and to determine if different suture sizes result in significant differences in tissue biomechanics and tissue reactivity following tissue closure. This study focuses on companion animal clinical and aims to explore the variations in tissue biomechanics and tissue reactivity after canine abdominal incision closure using commonly used USP 2-0, 3-0, and 4-0 suture threads. We hypothesize that smaller suture needles and threads can result in lower tissue reactions while providing sufficient mechanical stability. Thus, smaller suture needles and threads for abdominal wall closure represent a more promising surgical suturing option.

## 2. Method

### 2.1. Biomechanical study

For biomechanical testing, 14 samples of canine abdominal fascia muscles and skin were prepared. They were collected and frozen for a period over several months from dogs weighing 5–15 kg. Within 12 h of the initiation of the thawing process, the samples were divided and measured at room temperature. The original samples were cut into tissue blocks measuring 3 × 10 cm, with 3 cm parallel to the midline of the abdomen and 5 cm on each side of the midline. To prevent drying, the test samples were covered with moistened cloths. Prior to conducting biomechanical testing, the tissue blocks underwent incisions along the midline of the abdomen, then the incision was opposed randomly only with (a) simple interrupted suture pattern with USP 2-0 suture, (b) simple continuous suture pattern with USP 2-0 suture, (c) simple interrupted suture pattern with USP 3-0 suture, (d) simple continuous suture pattern with USP 3-0 suture, (e) simple interrupted suture pattern with USP 4-0 suture, (f) simple continuous suture pattern with USP 4-0 suture. Each suture combination treatment was performed for four times. All incisions were created and sutured by the same veterinary surgeon. The suturing technique utilized a small stitch (3–5 mm) with a suture length to incision length (SL:WL) ratio of 4:1. The control group samples did not undergo midline incision and suturing prior to the biomechanical testing.

**Figure 1:**
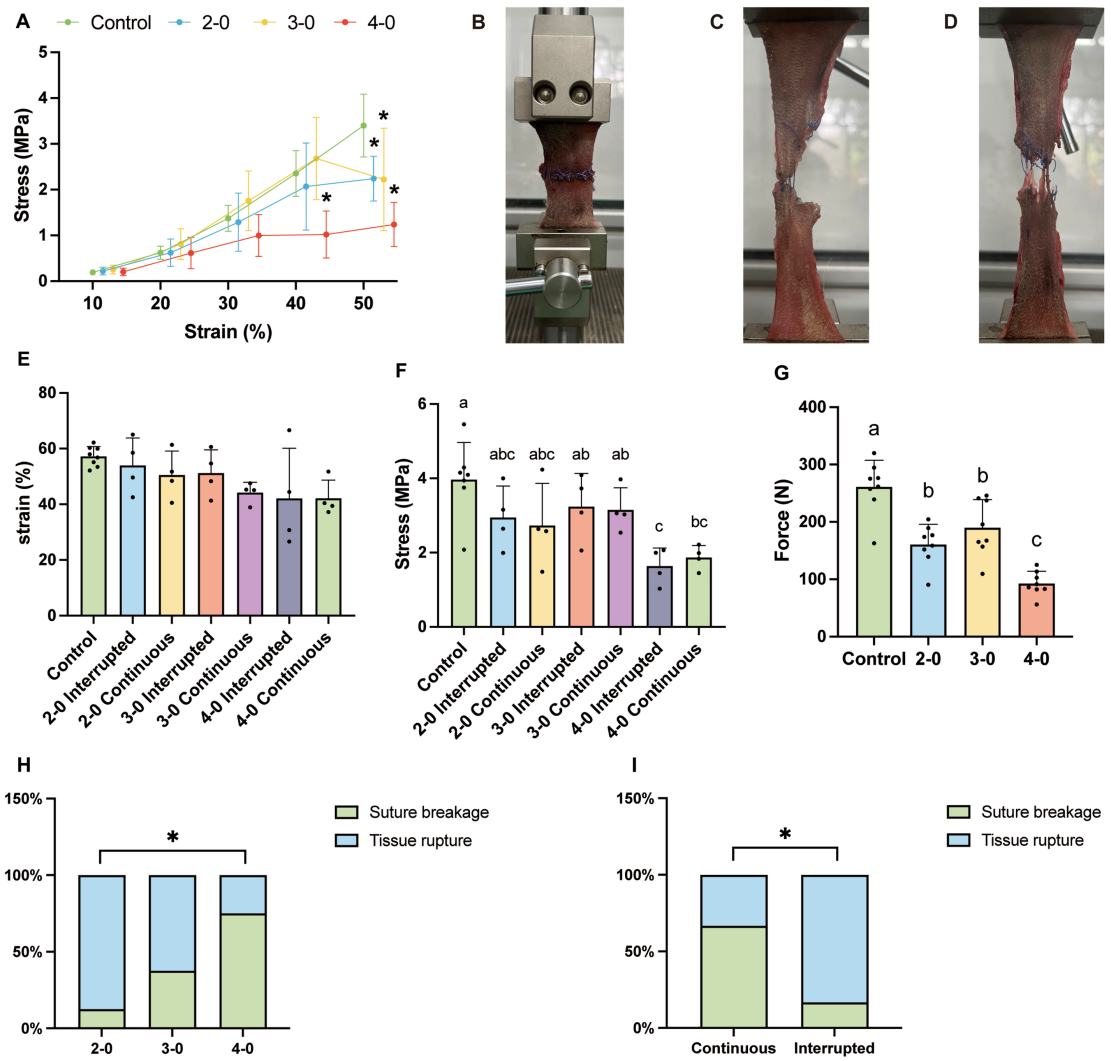
Uniaxial tensile testing of canine skin tissue *in vitro*. 2-0, 3-0, and 4-0 refer to USP 2-0 PGA, USP 3-0 PGA, and USP 4-0 PGA, respectively. “Interrupted” denotes simple interrupted sutures. “Continuous” represents simple continuous sutures. All abbreviations are the same as below. **(A)** Stress–strain curve. ^*^p < 0.05 (experimental groups vs. control group). **(B)** The initial state of the skin fixed to the tensile tester. **(C)** Suture breakage under high tension. **(D)** Tissue rupture at the site of skin sutures under high tension. **(E)** Tissue strain at the maximum tensile strength of the skin. **(F)** Stress value at the maximum tensile strength of the skin. **(G)** Maximum tensile strength of the skin. Two groups without any same letters indicate that there are significant differences, *p* < 0.05. **(H)** The suture failure endpoint with different sizes of suture needles and threads (Fisher’s exact test, ^*^p < 0.05). **(I)** The suture failure endpoint with different suture methods (Fisher’s exact test, ^*^p < 0.05).

The biomechanical experiments utilized an LLOYD LR10K plus mechanical testing machine equipped with a 1,000 N load cell. The test samples were secured to the tensile tester following the depiction in Figure 1B. In their initial position, the samples were kept in their natural extension, without the addition of extra tension. Sample thickness, clamping length, and width were measured using a vernier caliper. The measurement software applied a pre-load stress of 0.1 N/mm and recorded the corresponding stress values at 10%, 20%, 30%, 40%, and 50% strain, along with the stress and strain values at maximum tensile force. The stretching process involved documenting endpoint types, encompassing suture breakage, rupture of the sutured tissues, and other instances of tissue tearing.

### 2.2. Animals

This experiment involved a total of 27 adult beagles with an average weight of 13.4 ± 1.48 kg. All animal experiments were conducted at the Experimental Animal Center of Nanjing Agricultural University. The Animal Ethics Committee of Nanjing Agricultural University approved the experiment (Approval number: NJAU No. 20230123014), ensuring compliance with all relevant ethical regulations for animal testing and research. A pre-experimental period of 2 weeks was provided to allow the animals to acclimate to the environment. The animals were individually housed in cages under a 12 h light/12 h dark cycle within the animal facility. Each experimental dog received a complete commercial diet, and access to clean drinking water was available at all times. Caretakers monitored the dogs’ overall health and activity on a daily basis. Throughout the experiment, no unexpected events occurred that compromised the safety and well-being of the dogs. At the designated endpoints, the dogs were euthanized by intravenous injection of 200 mg/kg pentobarbital sodium solution in accordance with the 2020 AVMA Guidelines for the Euthanasia of Animals (30).

### 2.3. Animal protocol

To investigate whether there are significant differences in tissue reactions after suturing canine abdominal wall surgical incisions with different suture sizes, three types of sutures (USP 2-0, 3-0, and 4-0 PGA) produced by China HaiDiKe Company were selected for the experiment. The incisions in dogs were closed randomly only with (a) simple interrupted suture pattern with USP 2-0 suture, (b) simple continuous suture pattern with USP 2-0 suture, (c) simple interrupted suture pattern with USP 3-0 suture, (d) simple continuous suture pattern with USP 3-0 suture, (e) simple interrupted suture pattern with USP 4-0 suture, (f) simple continuous suture pattern with USP 4-0 suture. Each suture combination treatment was performed for four times.

Preoperative analgesia and sedation were managed by subcutaneous injection of meloxicam (0.2 mg/kg), intramuscular injection of bupropion (0.2 mg/kg), and dexmedetomidine (15 mcg/kg). Propofol (5 mg/kg) was injected intravenously to induce anesthesia in dogs. Airway access was established and anesthesia was maintained with isoflurane. Local anesthesia was performed by subcutaneous injection of 0.5% lidocaine at the surgical site. After anesthesia, sterile techniques were employed, and three intermittent incisions with a length of 3 cm were made along the midline of the abdomen, starting 3 cm posterior to the xiphoid process, with a spacing of 1–2 cm between each pair of incisions. Each incision was ensured to enter the abdominal cavity after dissecting the linea alba. These intermittent incisions aimed to avoid mutual interference of inflammation caused by different suture materials in different incisions after closure. Each experimental animal’s three incisions were sutured by the same surgeon using either simple interrupted or simple continuous suturing. Suturing followed a small stitch (3–5 mm) technique with an SL:WL of 4:1. Different suture specifications were alternated among different animals for incisions in different locations to minimize the impact of wound position on healing. Meloxicam (0.1 mg/kg) was injected subcutaneously daily for 5 consecutive days after surgery.

During the experiment, the experimental animals were observed daily for signs of wound dehiscence, incisional hernia, wound infection, and the appearance of the skin after suturing. Skin samples were collected at 1 day, 7 days, and 14 days after suturing. Linea alba samples were collected at 1 day, 7 days, and 3 months after suturing. Each sample was divided into aliquots and stored in freezing tubes and tissue fixatives for the detection of inflammatory factor expression levels and histological evaluation, respectively.

### 2.4. Total mRNA extraction and quantitative RT-PCR

Samples were collected and immediately frozen in liquid nitrogen and homogenized in TRIzol reagent (Angle Gene) to obtain mRNA. RNA was extracted and purified from abdominal wall wounds using chloroform, isopropanol, and ethanol. RNA purity and quantity were assessed using the NANODROP ONE system (Thermo). In accordance with the manufacturer’s protocol, the total RNA (100 μg) was reverse transcribed into cDNA using a reverse transcription enzyme kit (TransGen Biotech). By using the PerfectStart Uni RT&qPCR Kit (TransGen Biotech) and the 7300 Real-Time PCR System, the absorbance of the resulting cDNA was analyzed by qPCR. The primer sequences for real-time fluorescence quantitative PCR were designed using PrimerBLAST software (NCBI), and their sequences are shown in Table 1. Comparative Ct (∆∆Ct) analysis of the tissues’ mRNA expression was performed with normalization based on the average expression of the GAPDH reference mRNA in the tissues.

**Table 1:** Primer sequence of canine genes examined by quantitative real-time PCR.

Target gene	Primer sequence
IL-1β (forward primer)	5′-CTGCCAAGACCTGAACCAC-3′
IL-1β (reverse primer)	5′-AGCTACAATGACTGACACGAA-3′
IL-6 (forward primer)	5′-AGATTCCAAGGATGATGCCAC-3′
IL-6 (reverse primer)	5′-ACATCTCCTTTCTCAGTGCAGA-3′
TNF-ɑ (forward primer)	5′-GACAAAGGTCAACCTACT-3′
TNF-ɑ (reverse primer)	5′-GCAAAGTCCAGATAGTTAG-3′
GAPDH (forward primer)	5′-ATGGTGAAGGTCGGAGTGAAC-3′
GAPDH (reverse primer)	5′-CCACAACATACTCAGCACCAG-3′

### 2.5. Enzyme-linked immunosorbent assay

Tissue samples for protein content analysis were rapidly frozen in liquid nitrogen immediately after harvesting. The frozen tissues were ground into powder under liquid nitrogen cooling. A appropriate amount of PBS was added to the tissue powder and thoroughly homogenized. After homogenization, the suspension was centrifuged (20 min, 3,000 rpm), and the supernatant was transferred to a new tube. The protein concentration was determined using the Bradford protein assay (BioRad). The concentration of IL-1β was measured using the Canine IL-1β ELISA kit (Angle Gene). The concentration of IL-6 was measured using the Canine IL-6 ELISA kit (Angle Gene). The concentration of TNF-α was measured using the Canine TNF-α ELISA kit (Angle Gene). A total protein amount of 10 μg per well was used for the detection, following the manufacturer’s protocol. Normalization was performed for each protein concentration.

### 2.6. Histology

After sampling, the specimens intended for histological examination were fixed in 4% paraformaldehyde, embedded in paraffin, and sectioned at a thickness of 4 mm. The sections were stained with hematoxylin and eosin (H&E) and then dehydrated and coverslipped. The overall condition of the sections and the position of the sutures were observed using an optical microscope (LEICA DM500) at a magnification of 100×. Photographs were taken by a pathologist unaware of the experimental groups at four different locations (upper, lower, left, and right) around the suture holes on each slide using a magnification of 400×. According to the relevant provisions in ISO10993, the degree of inflammation and tissue reactivity were evaluated. The degree of inflammation scoring included counts of polymorphonuclear cells, lymphocytes, plasma cells, macrophages, and giant cells, as well as the degree of necrosis. The tissue reactivity score was calculated by adding the inflammation score (multiplied by 2), neovascularization score, fibrosis score, and fat infiltration score. Cell counts were scored based on the number of cells per high-power field at 400× magnification: absent (0), 1–5/HPF (1), 5–10/HPF (2), severe infiltration (3), and full field (4). Scores for necrosis, neovascularization, fibrosis, and fat infiltration were categorized as absent (0), mild (1), moderate (2), moderate-to-severe (3), and severe (4).

### 2.7. Statistical analysis

The data were presented as mean ± SD. Fisher’s exact test was used to analyze the differences in the occurrence rates of the biomechanical endpoints. Mann–Whitney *U* test and Wilcoxon test of the stepwise method were used for the remaining data. Statistical significance was defined as *p* < 0.05. In the figures, two groups without any same letters indicate that those have significant differences. All the statistical analyses mentioned above were performed using SPSS (version 26.0.0.2, IBM), and the figures were generated using GraphPad Prism 9 (version 9.5.1).

## 3. Result

### 3.1. Biomechanical test results

Skin biomechanical tests showed no significant stress differences between the experimental and control groups below 30% strain. However, at 40% strain, the USP 4-0 PGA suture group showed significantly lower stress compared to the other experimental groups and the control group, indicating suture support failure in the tissue. The USP 3-0 and 2-0 sutures lost their effective support at a 50% strain (Figure 1A), suggesting suture failure in small-diameter sutures with low tissue deformation. But it is important to note that a 30% abdominal wall deformation is uncommon in clinical practice. The strain in the USP 4-0 suture group was lower at maximum tension, but no significant differences were observed, nor between interrupted and continuous sutures (Figure 1E). Furthermore, the USP 4-0 sutured skin had significantly lower maximum tensile strength with a mean value of 92.5 N (Figure 1G). However, regardless of suture diameter, no significant difference in maximum stress was found between continuous and interrupted sutures (Figure 1F), suggesting similar tissue support capabilities. Tensile testing after suturing identified two endpoints: suture breakage (Figure 1C) and tissue rupture at the suture site (Figure 1D). Larger-diameter sutures caused tissue tearing under high tension, while smaller-diameter sutures were prone to suture breakage (Figure 1H). Interestingly, under high-tension conditions, continuous sutures had a higher likelihood of suture breakage, while interrupted sutures resulted in more tissue rupture (Figure 1I).

**Figure 2:**
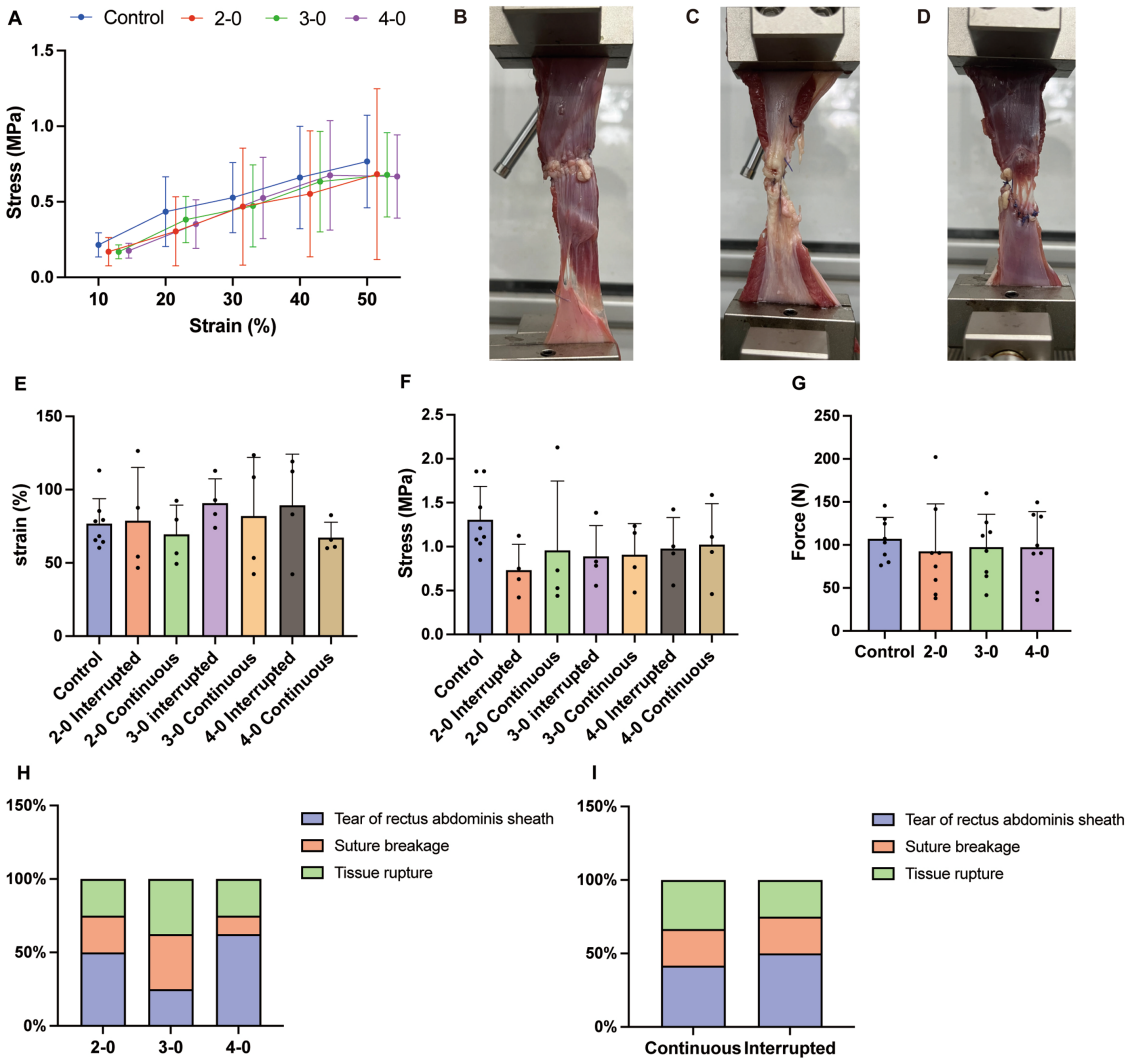
Uniaxial tensile testing of canine fascia and muscle tissue *in vitro*. **(A)** Stress–strain curve. **(B)** Rectus abdominis sheath tear under high tension **(C)** Suture breakage. **(D)** Tissue rupture at the suture site. **(E)** Strain at the maximum tensile strength. **(F)** Stress value at the maximum tensile strength. **(G)** Maximum tensile strength. **(H)** The suture failure endpoint with different sizes of needles and threads sutured linea alba (Fisher’s exact test). **(I)** The suture failure endpoint with different suturing methods sutured linea alba (Fisher’s exact test).

Muscle biomechanical test showed no significant stress differences between experimental and control groups below 50% strain (Figure 2A). Maximum tensile strength (Figure 2G), corresponding stress (Figure 2F), and strain (Figure 2E) during muscle elongation were also similar between groups. Similar to the skin tissue, muscle elongation testing revealed suture breakage (Figure 2C) and tissue rupture at the suture site (Figure 2D). However, unlike the skin tissue, half of the muscle tests resulted in non-sutured fascial tearing (Figure 2B). Regardless of suture diameter and technique, there were no significant differences in the occurrence rates of test endpoints (Figures 2H,I). These findings suggest no significant differences in mechanical properties of the abdominal wall after canine linea alba suturing using USP 2-0, 3-0, and 4-0 PGA sutures. The sutured abdominal wall exhibited similar mechanical properties to the intact wall under extreme tension conditions.

### 3.2. Observation of the appearance of the skin incision after suturing

**Figure 3:**
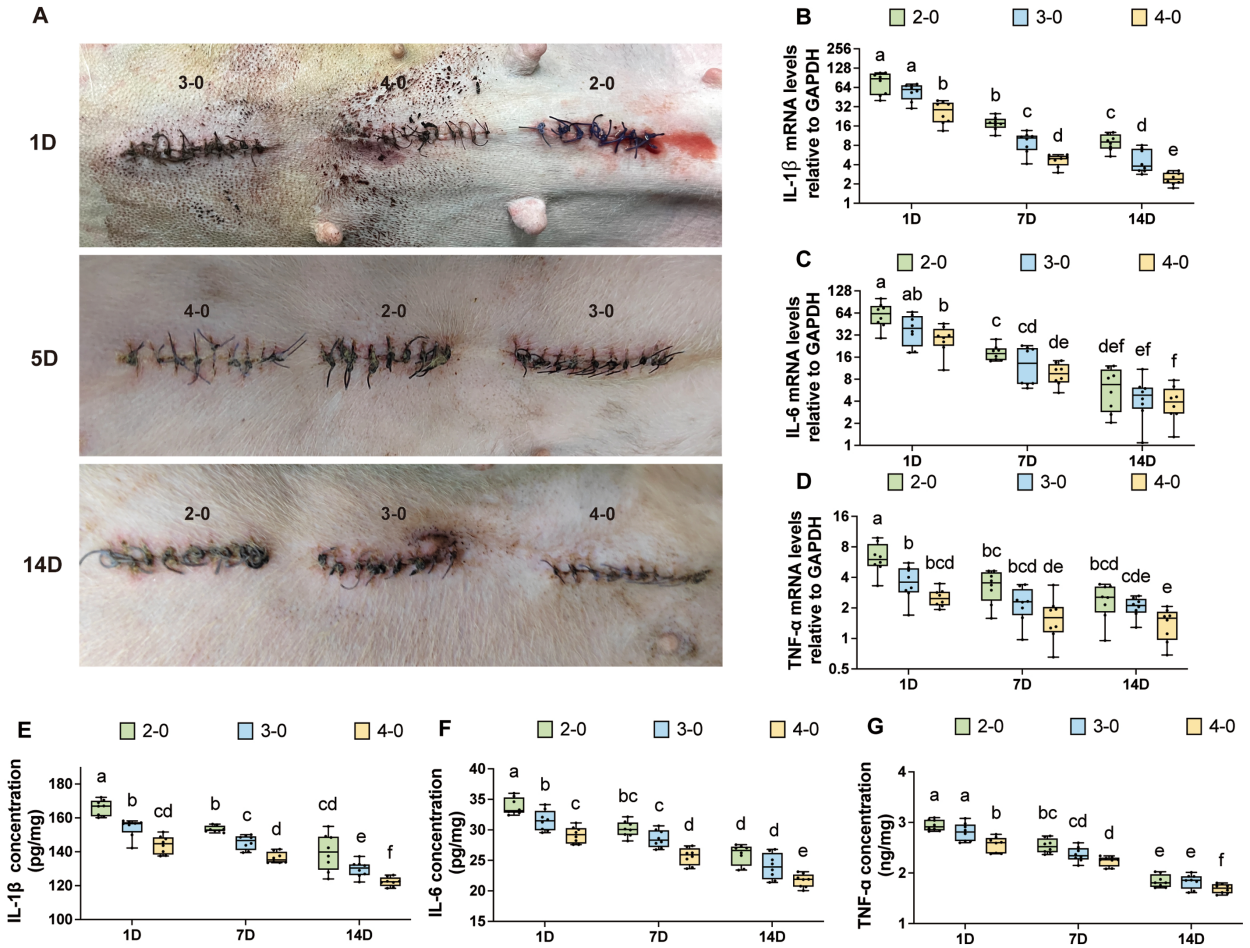
Evaluation of inflammatory cytokine after canine skin suture. **(A)** The appearance of the skin incision after suturing, with different rows representing 1, 5, and 14 days post-suturing, and the USP suture gauge indicated above each incision. **(B–D)** mRNA expression levels of IL-1β, IL-6, and TNF-α after skin suturing (USP 2-0 PGA vs. USP 3-0 PGA vs. USP 4-0 PGA). **(E–G)** Protein expression levels of IL-1β, IL-6, and TNF-α after skin suturing (USP 2-0 PGA vs. USP 3-0 PGA vs. USP 4-0 PGA).

No animals showed signs of infection, wound dehiscence, or incisional hernia throughout the experiment. Figure 3A shows the postoperative appearance of the skin incision. Mild redness and exudation were observed in incisions during the initial week, regardless of suture diameter. However, at 1–2 weeks after closure, the USP 2-0 and 3-0 suture groups had a higher incidence of redness and scab formation, while the USP 4-0 group had milder redness and less frequent scab formation.

### 3.3. Small-diameter sutures significantly reduce skin tissue reactivity after suturing

mRNA expression levels of IL-1β and IL-6 gradually decreased after suturing but remained significantly higher than the control group (Supplementary Figures S1A,B). TNF-α mRNA expression showed no significant differences at 7 and 14 days after suturing (Supplementary Figure S1C). Figures 3B–D show variations in mRNA levels among different suture diameter groups. Except for IL-6 expression at 14 days, which had no significant difference between USP 2-0 and 4-0 groups, inflammatory factor levels were consistently higher in the USP 2-0 group at other time points. Additionally, IL-1β mRNA expression in the USP 2-0 group at 14 days remained significantly higher than the USP 4-0 group at 7 days. The USP 3-0 group had lower IL-1β expression than USP 2-0 and higher than USP 4-0 at 14 days. Suture methods did not significantly affect inflammatory factor expression (Supplementary Figures S1D–F) since simple interrupted sutures position knots outside the skin.

Protein expression levels of IL-1β, IL-6, and TNF-α in the skin followed a comparable pattern to mRNA expression. Over time, inflammatory cytokine levels gradually decreased but remained significantly higher than the control group (Supplementary Figures S2A–C). At each time point after suturing, the USP 4-0 PGA suture group showed significantly lower protein expression levels of IL-1β, IL-6, and TNF-α compared to the USP 2-0 PGA suture group (Figures 3E–G). Additionally, both simple continuous and simple interrupted suturing had no significant impact on protein expression levels of these inflammatory cytokines at any time point after skin suturing (Supplementary Figures S2D–F).

**Figure 4:**
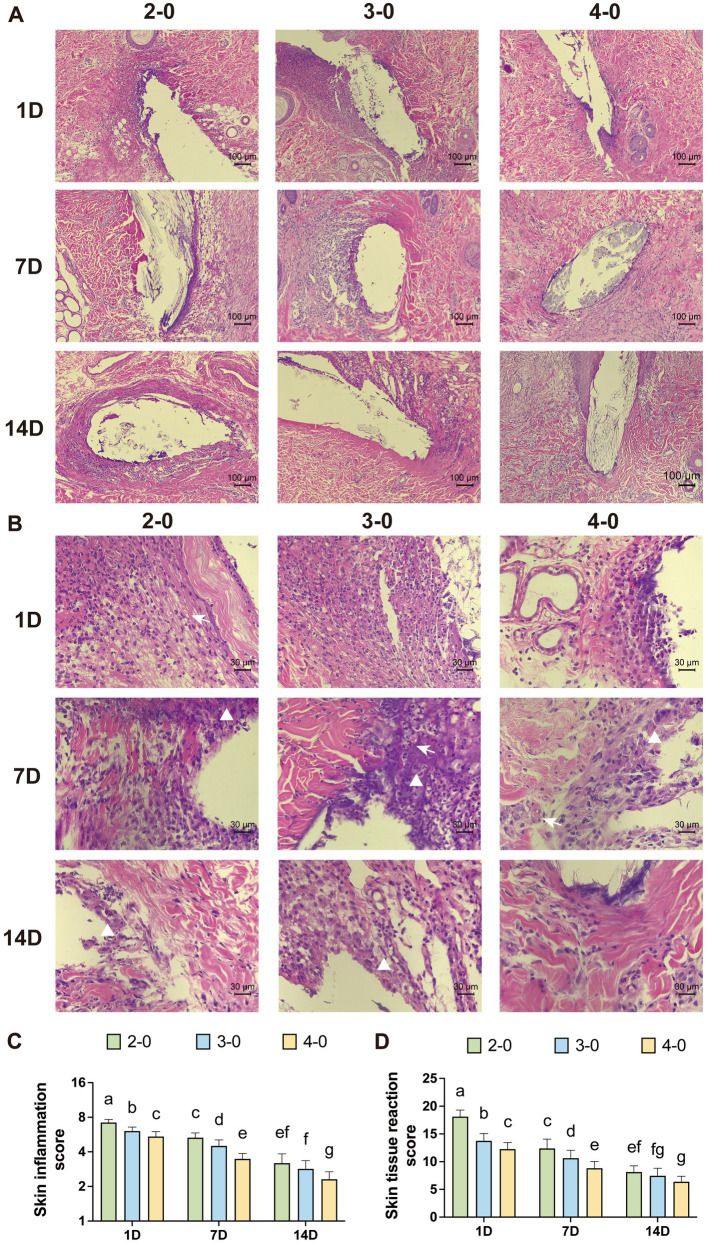
Evaluation of Histopathological after canine skin suture. **(A)** Representative H&E-stained sections after skin sutured (100× magnification). **(B)** Representative H&E-stained sections after skin sutured (400× magnification). Different rows representing 1, 7, and 14 days post-suturing, and different columns representing the suture gauge used: USP 2-0, 3-0, and 4-0 PGA. White triangles mark macrophage infiltration. White arrows mark neovascularization. **(C)** Inflammation score around the suture threads. **(D)** Tissue reactivity scores around the suture threads. Two groups without any same letters indicate that there are significant differences, *p* < 0.05.

Under 100× magnification, the intense purple-colored inflammatory infiltrate was observed around larger suture needle holes on the first-day post-surgery. At 400× magnification, numerous polymorphonuclear cells were visible. On the 7th day post-surgery, the deep staining area around suture holes in the USP 4-0 group was relatively smaller. Polymorphonuclear cell infiltration decreased, but severe infiltration remained in the USP 2-0 group. At this point, an increase in macrophages and giant cells was observed. Mild neovascularization was observed in the skin tissue. After 14 days, the USP 2-0 group showed prominent fibrous capsule formation around suture holes (Figures 4A,B). All groups had mild to moderate polymorphonuclear cell infiltration, with macrophages predominantly around suture holes. Inflammation and tissue reactivity scores decreased with time, with significantly higher scores in the USP 2-0 group compared to the USP 4-0 group (Figures 4C,D).

### 3.4. Small-diameter sutures significantly reduce fascial muscle tissue reactivity after suturing

**Figure 5:**
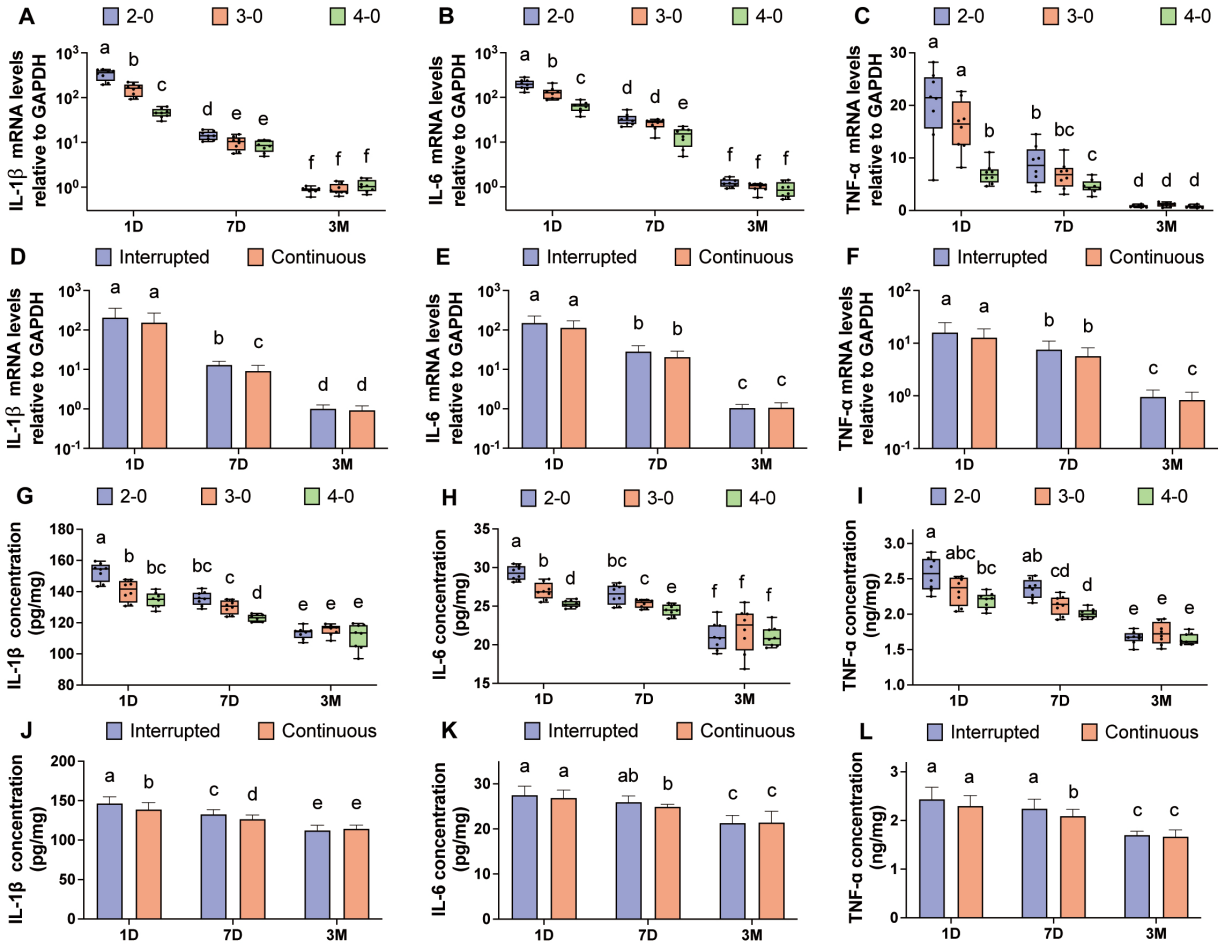
Evaluation of inflammatory cytokine after canine linea alba suture. **(A–C)** mRNA expression levels of IL-1β, IL-6, and TNF-α in the fascia and muscle tissue (USP 2-0 PGA vs. USP 3-0 PGA vs. USP 4-0 PGA). **(D–F)** mRNA expression levels of IL-1β, IL-6, and TNF-α in the fascia and muscle tissue (simple interrupted sutures vs. simple continuous sutures). **(G–I)** Protein expression levels of IL-1β, IL-6, and TNF-α in the fascia and muscle tissue (USP 2-0 PGA vs. USP 3-0 PGA vs. USP 4-0 PGA). **(J–L)** Protein expression levels of IL-1β, IL-6, and TNF-α in the fascia and muscle tissue (simple interrupted sutures vs. simple continuous sutures).

Three months after linea alba sutures, complete suture absorption was observed, and scar examination revealed no significant differences in mRNA expression levels of IL-1β, IL-6, and TNF-α compared to the control group (Supplementary Figures S3A–C). Figures 5A–C illustrate variations in mRNA levels among experimental groups. At 3 months, no significant differences were observed among suture line groups. However, on postoperative day 1 and day 7, USP 2-0 sutures showed significantly higher mRNA expression levels of IL-1β, IL-6, and TNF-α compared to USP 4-0 sutures. Unlike skin suturing, simple interrupted suturing exhibited higher IL-1β mRNA expression levels compared to simple continuous suturing at 7 days after linea alba closure (Figure 5D).

Three months after suturing, no significant differences in protein expression levels were found compared to the control group (Supplementary Figures S3D–F). On postoperative day 1 and day 7, the USP 4-0 PGA suture group showed significantly lower protein expression levels of IL-1β, IL-6, and TNF-α compared to the USP 2-0 PGA suture group (Figures 5G–I). Simple continuous suturing resulted in lower IL-1β protein expression on the first day after suturing (Figure 5J). However, at 7 days, simple interrupted suturing led to higher levels of IL-1β and TNF-α protein expression (Figures 5J,L).

**Figure 6:**
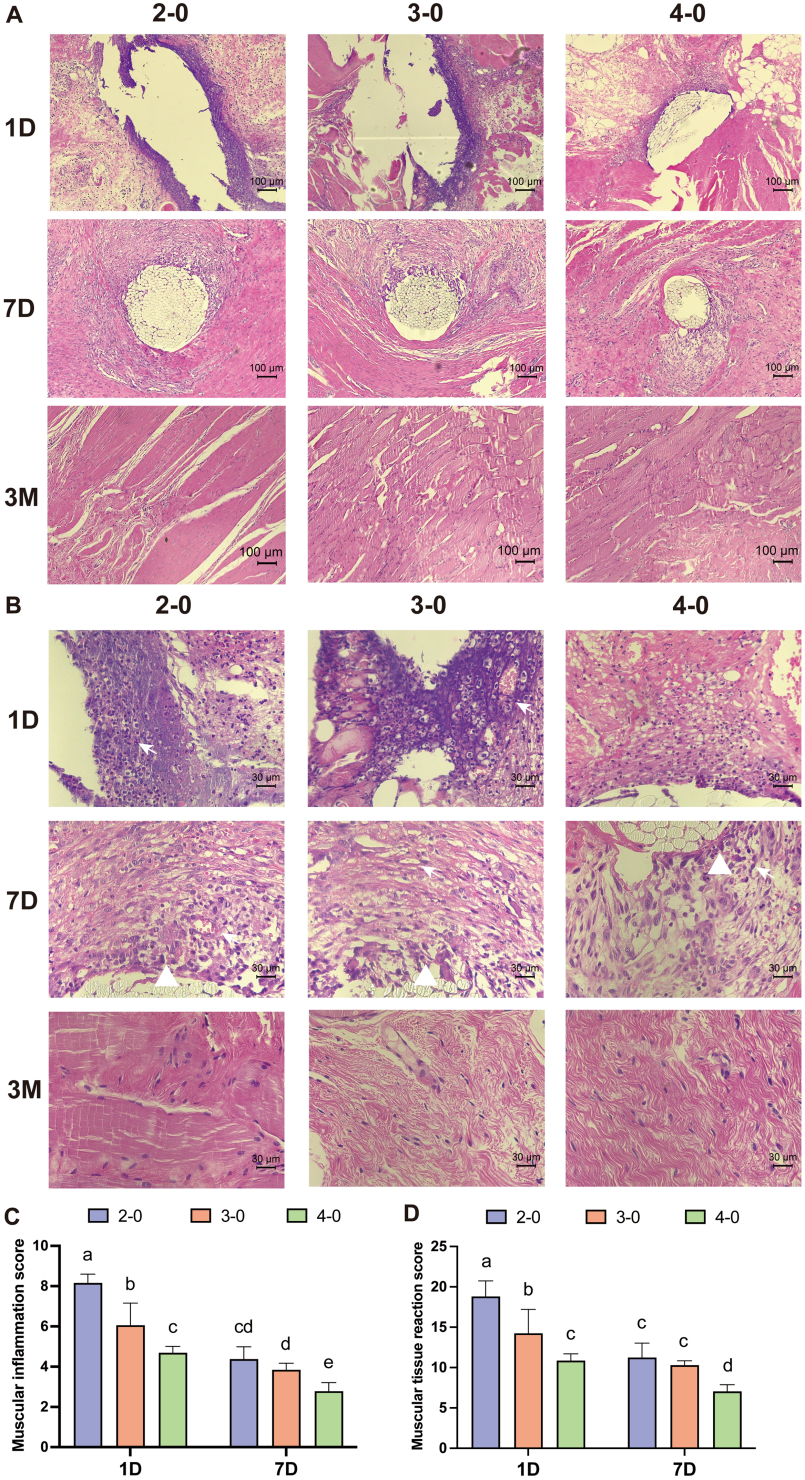
Evaluation of histopathological after canine linea alba suture. **(A)** Representative H&E-stained sections after linea alba sutured (100× magnification). **(B)** Representative H&E-stained sections after linea alba sutured (400× magnification). Different rows representing 1, 7, and 14 days post-suturing, and different columns representing the suture threads used: USP 2-0, 3-0, and 4-0 PGA. White triangles mark macrophage infiltration. White arrows mark neovascularization. **(C)** Inflammation score around the suture threads. **(D)** Tissue reactivity scores in the surrounding tissue. Two groups without any same letters indicate that there are significant differences, *p* < 0.05.

Under 100× magnification, HE-stained sections of the fascia muscle tissue showed pronounced deep purple inflammatory infiltrates surrounding larger suture needle holes on the first day after the operation. At 400× magnification, numerous polymorphonuclear cells and lymphocytes were observed infiltrating the field of view. Muscle tissue displayed necrotic tissue, neovascularization, and varying effects of different suture diameters on the surrounding tissue on the seventh day. After 3 months, no significant differences were observed among the suture groups in the tissue sections (Figures 6A,B). Inflammatory and tissue reaction scores decreased over time, with significantly higher scores for the USP 2-0 suture group compared to the USP 4-0 suture group (Figures 6C,D). No significant differences in fascia muscle tissue fibrosis caused by different suture diameters were observed during the early post-suturing phase.

## 4. Discussion

Normal wound healing is the foundation of successful surgery. However, poor abdominal wall healing remains a common complication of abdominal surgery. According to statistics, the incidence of postoperative incisional hernia in humans is 12.8% (2), and the incidence of wound dehiscence reaches 5.5% (3). Although explicit statistical research is lacking, delayed wound healing and poor healing are commonly observed postoperative complications in veterinary clinical practice. Multiple research studies have now published guidelines regarding abdominal wall suturing, which recommend continuous suturing (31), close stitch intervals (28), SL:WL greater than 4 (32), and the use of antimicrobial sutures (33). Suturing aims to maintain a certain level of tension in the wound using sutures, facilitating wound edge approximation and accelerating healing. However, there is a paradox in that sutures themselves, as foreign materials, can worsen the inflammatory response in the wound, leading to poor healing (24). Currently, several studies are focused on developing novel suture materials or coating existing sutures with new materials to reduce tissue reactivity following suture implantation. Early research has found that surgeons may generally overestimate the tension required for tissue suturing, indicating that smaller sutures with lower tensile strength may also fulfill the tension requirements after suturing and cause less tissue reaction.

Insufficient suture tension is well-known as one of the major causes of suture failure (34). Due to the uncertainty regarding the tension requirements at the suture site, many surgeons tend to use larger gauge sutures with high tensile strength for wound closure. However, a previous study on the tension of the canine abdominal wall in 59 clinical surgeries revealed that the median tension at the midpoint of muscle incisions was only 1.51 N, with a maximum tension of only 4.23 N, while the median tension for skin incisions was only 0.67 N (27). These findings indicate that the actual tension required by the tissues during surgical suturing may be significantly lower than anticipated by surgeons. A single USP 4-0 PGA suture has a tensile strength of at least 12 N, indicating the need for optimizing the selection of suture material for abdominal wall closure in canine surgery. In this study, although the maximum tensile strength of USP 4-0 PGA sutures for closing canine skin was significantly lower than that of other groups, its minimum value far exceeded the measured skin closure tension requirements in previous experiments. In the linea alba closure experiment, there was no significant difference in maximum tensile strength or maximum stress between the groups, and in nearly half of the test groups, the endpoint of the tensile test was the tearing of the fascia of the rectus abdominis muscle rather than the rupture at the suture site. Our data suggests that even when using USP 4-0 PGA sutures for linea abla, their mechanical properties are very similar to the intact linea abla area. Intra-abdominal pressure is an important component of abdominal wall tension, and studies have shown a linear positive correlation between intra-abdominal pressure and abdominal wall tension (35). Commonly intra-abdominal pressure in humans does not exceed 11 kPa (36, 37), and further research indicates that under maximum physiological load, the strain in the linea alba region is approximately 7%–12% (38). In this experimental model, no significant differences were observed among the groups in terms of skin strain below 30% and muscle strain below 50%, suggesting that under physiological conditions, USP 2-0, 3-0, and 4-0 PGA sutures are all capable of providing sufficient support for the incision after suturing the canine abdominal wall.

The objective fact is that finer sutures have lower tensile strength, so some surgeons believe that using finer sutures such as USP 4-0 for wound closure may lead to suture breakage. Based on the findings of this study, in routine low-tension surgical wound closure in dogs, the breakage of USP 4-0 sutures may be due to inappropriate additional tension applied by the surgeon during suturing and knotting. Inaccurate assessment of tissue tension by surgeons can not only cause unnecessary suture breakage but also result in high-tension closure. High-tension closure can lead to compromised blood supply (39) and disrupted collagen distribution (40) at the suture site, increasing the incidence of postoperative complications (41). Surgeons should minimize additional tension while ensuring alignment of the incision edges. For canine surgical incisions without signs of high tension, such as significant margin separation, using finer USP 4-0 sutures for abdominal wall closure can maintain the stability of the incision. The high tensile strength provided by larger diameter sutures such as USP 2-0 may not be necessary.

Inflammation is a vital stage in the healing process, essential for hemostasis and initiation of non-specific immune responses (42). Excessive inflammation impedes normal healing, leading to delayed healing and excessive scar formation (42). In this work, larger-diameter sutures consistently showed higher levels of inflammatory factors and tissue reactions at various time points. Even all suture sizes completely degraded in the muscle after 3 months of suturing, and no significant differences were observed in the expression levels of inflammatory factors and tissue histological scores. However, the impact of different suture sizes on early postoperative tissue cannot be ignored. In fact, inflammation plays a crucial role in early infection prevention in wounds, while rapid suppression of inflammatory cells and downregulation of pro-inflammatory cytokines promote early tissue repair and subsequent tissue remodeling, accelerating wound healing, especially in non-infectious wounds such as surgical incisions (42). Neutrophil depletion (18) or macrophage knockout mice (19) actually exhibited accelerated healing and reduced scarring due to the absence of these inflammatory cells. Substances such as curcumin (43) and whey protein (44) improve wound healing by reducing the expression of pro-inflammatory cytokines such as IL-1β, IL-6, and TNF-α during the skin wound healing process. Yaman et al. (45) demonstrated that orally administered anti-inflammatory agent resveratrol in rats decreased abdominal wall inflammation levels and increased abdominal wall tensile strength. In the present study, the level of inflammation after tissue closure with USP 4-0 PGA sutures was significantly lower than that with USP 2-0 PGA sutures, indicating that the difference in suture diameter causing variation in foreign materials implantation can significantly alter the degree of tissue inflammatory response. Therefore, selecting finer sutures like USP 4-0 PGA for closure canine abdomen may be beneficial for tissue healing while maintaining sufficient tensile strength.

The suture knot is an integral part of a complete closure and, as an additional foreign material apart from the suture loop, it is also expected to elicit corresponding inflammatory responses. The results of this experiment demonstrate that, during linea alba suturing, interrupted sutures can induce greater expression of inflammatory factors compared to continuous sutures. However, their impact on tissue inflammation is relatively weaker compared to the choice of suture size. This finding is similar to the study by Van et al., who found that suture size and material are independent factors influencing tissue reactivity, while the suture knot has a minimal effect on tissue response (46). In this study, there were no significant differences in the mechanical properties of canine abdominal wall tissue between continuous and interrupted sutures under *ex vivo* conditions. However, on the other hand, in mice at 14 days postoperative, continuous sutures exhibited significantly higher abdominal wall mechanical strength compared to interrupted sutures, provided that the suture length-to-wound length ratio was greater than 4 (47). Furthermore, continuous sutures required less time to complete (48). Therefore, continuous sutures may have advantages over interrupted sutures in abdominal wall closure.

Nevertheless, there are certain limitations to our study. Firstly, although we have identified the advantages of suturing with small-diameter threads, the sample size was limited to experimental animals, and further validation is required through larger-scale clinical trials. Secondly, the *ex vivo* tissue strain testing conducted in this study employed a uniaxial testing method, which imposed only transverse pre-stress on the tissue prior to testing. However, in reality, the abdominal wall tissue is also influenced by longitudinal stress, thereby resulting in potential differences between the tissue’s mechanical properties measured in this experiment and *in vivo* conditions.

In conclusion, this study confirms that USP 4-0 PGA sutures are suitable for suturing the general canine abdominal wall, including the linea alba and skin, while satisfies the tension requirements. Additionally, reducing the suture diameter significantly decreases tissue reactivity, regardless of whether it is the linea alba or skin tissue. Suturing the canine abdominal wall with USP 4-0 PGA sutures exhibits minimal tissue reactivity and provides sufficient postoperative tissue mechanical stability. Surgeons should select the smallest suture size that meets the tension requirements when suturing abdominal wall incisions, aiming to minimize additional tissue inflammation and trauma.

## Data availability statement

The raw data supporting the conclusions of this article will be made available by the authors, without undue reservation.

## Ethics statement

The animal study was approved by the Animal Ethics Committee of Nanjing Agricultural University. The study was conducted in accordance with the local legislation and institutional requirements.

## Author contributions

SL: Data curation, Visualization, Writing – original draft. YG: Data curation, Writing – review & editing. XZ: Formal analysis, Writing – review & editing. DL: Investigation, Writing – review & editing. ZZ: Conceptualization, Resources, Writing – review & editing.

## Supplementary material

SUPPLEMENTARY FIGURE S1 The mRNA expression levels of inflammatory factors after canine skin sutured. **(A–C)** mRNA expression levels of IL-1β, IL-6, and TNF-α at 1, 7, and 14 days post-suturing. **(D–F)** mRNA expression levels of IL-1β, IL-6, and TNF-α after canine skin suturing, comparing simple interrupted sutures and simple continuous sutures.

SUPPLEMENTARY FIGURE S2 The protein expression levels of inflammatory factors after canine skin sutured. **(A–C)** Protein expression levels of IL-1β, IL-6, and TNF-α at 1, 7, and, 14 days post-suturing. **(D–F)** Protein expression levels of IL-1β, IL-6, and TNF-α after skin suturing, comparing simple interrupted sutures and simple continuous sutures.

SUPPLEMENTARY FIGURE S3 The expression levels of inflammatory factors in the fascia and muscle tissue after canine linea alba sutured. **(A–C)** mRNA expression levels of IL-1β, IL-6, and TNF-α in the fascia and muscle tissue at 1 day, 7 days, and 3 months post linea alba sutured. **(D–F)** Protein expression levels of IL-1β, IL-6, and TNF-α in the fascia and muscle tissue at 1 day, 7 days, and 3 months post linea alba sutured.
