# Supplementary figures and images for: Biomechanical and tissue reaction: the effects of varying sutures size on canine abdominal wall stitching
Source: Front Vet Sci. 2023 Nov 10;10:1254998. doi: 10.3389/fvets.2023.1254998 (PMC10667435; doi:10.3389/fvets.2023.1254998)

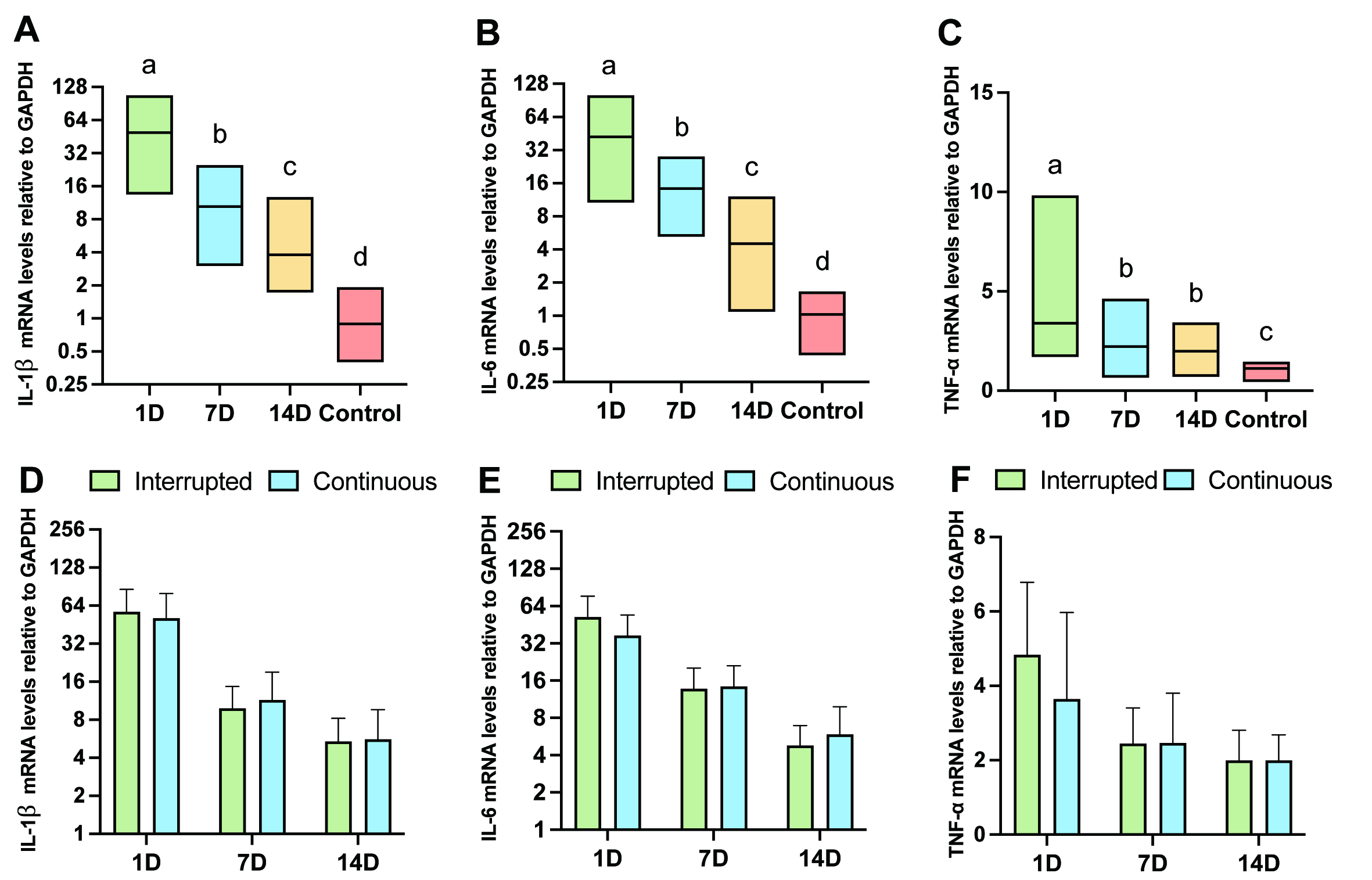

Supplement: SUPPLEMENTARY FIGURE S1 — The mRNA expression levels of inflammatory factors after canine skin sutured. (A–C) mRNA expression levels of IL-1β, IL-6, and TNF-α at 1, 7, and 14 days post-suturing. (D–F) mRNA expression levels of IL-1β, IL-6, and TNF-α after canine skin suturing, comparing simple interrupted sutures and simple continuous sutures. [file Image_1.JPEG]

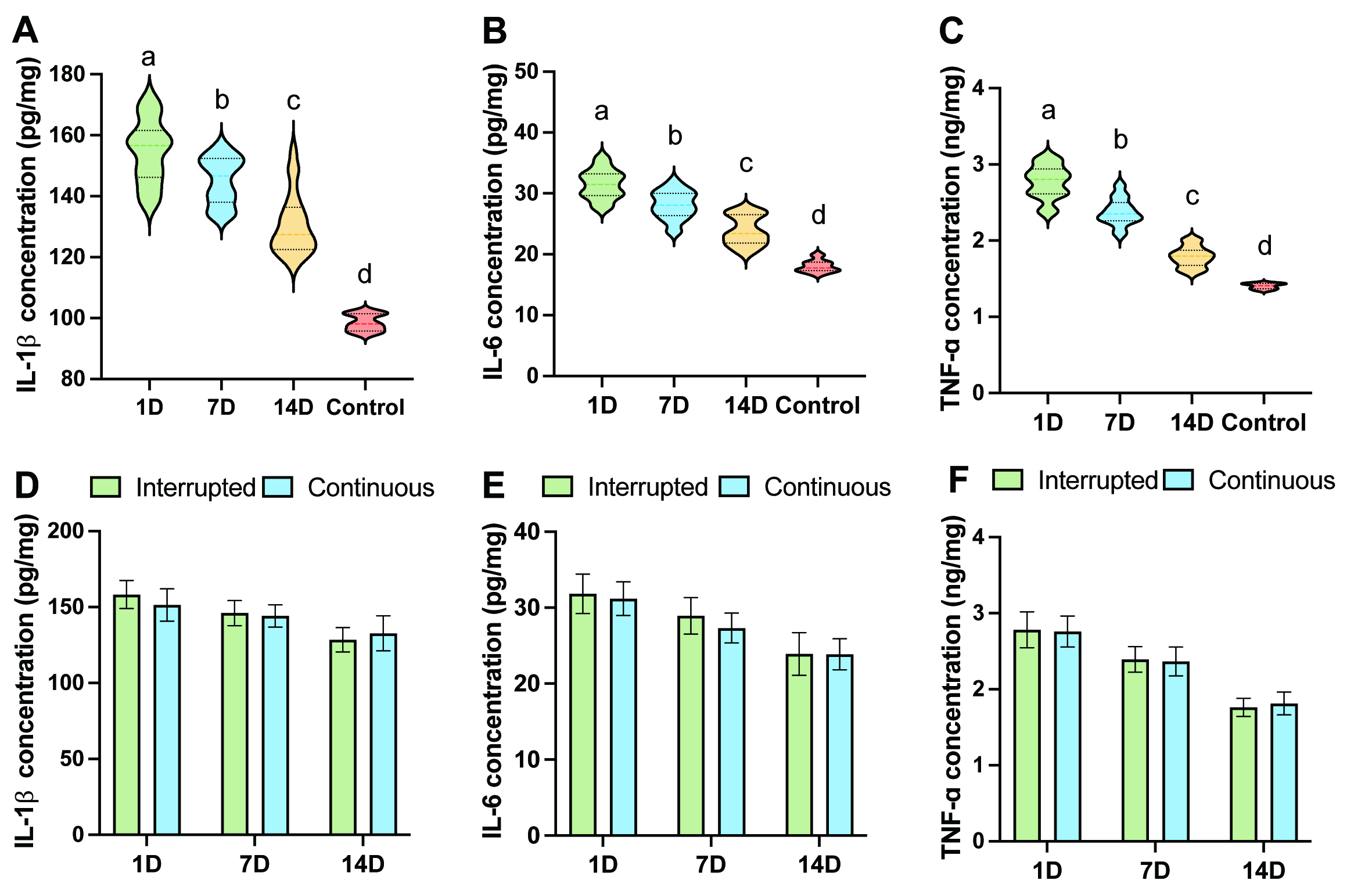

Supplement: SUPPLEMENTARY FIGURE S2 — The protein expression levels of inflammatory factors after canine skin sutured. (A–C) Protein expression levels of IL-1β, IL-6, and TNF-α at 1, 7, and, 14 days post-suturing. (D–F) Protein expression levels of IL-1β, IL-6, and TNF-α after skin suturing, comparing simple interrupted sutures and simple continuous sutures. [file Image_2.JPEG]

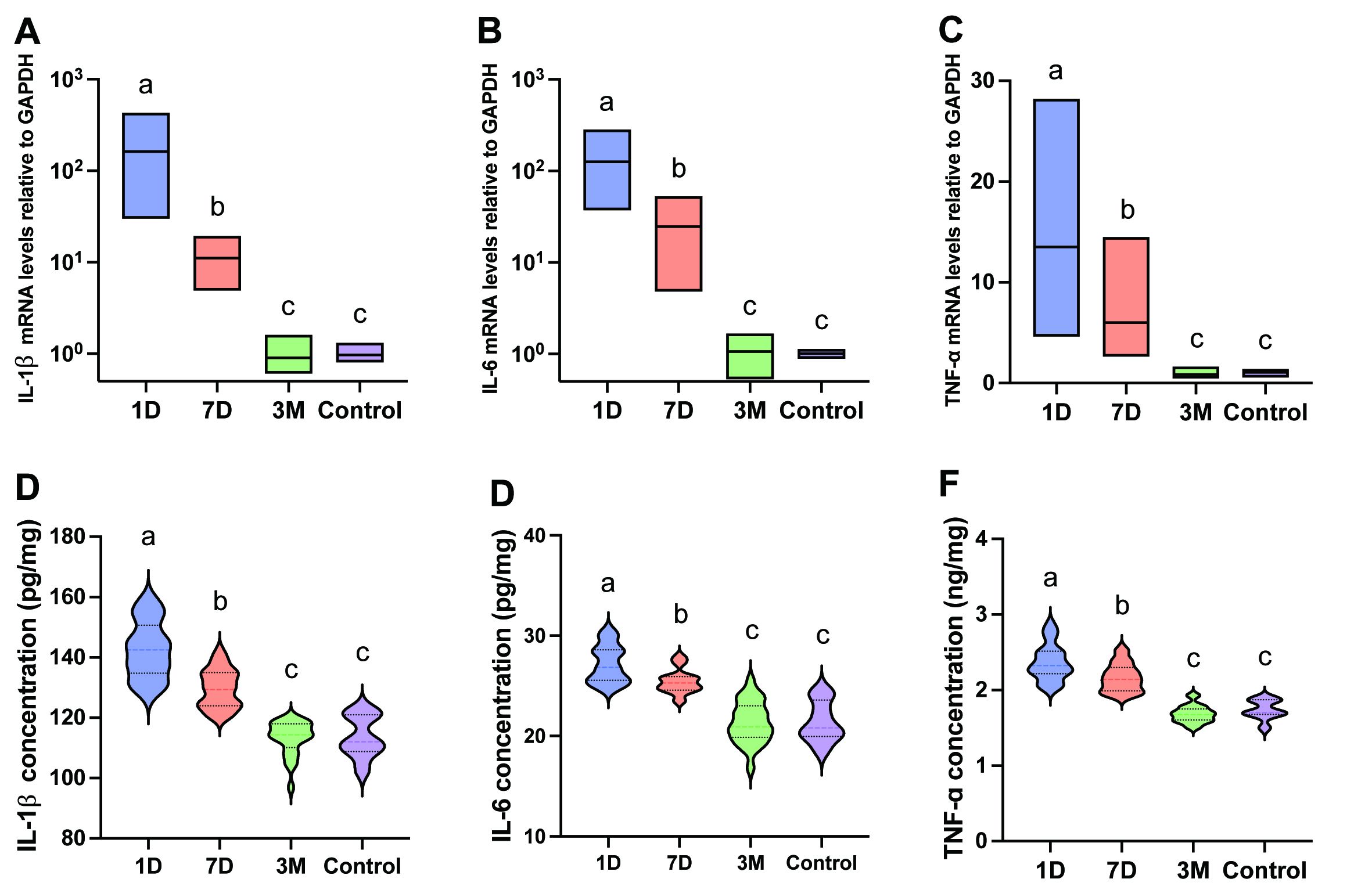

Supplement: SUPPLEMENTARY FIGURE S3 — The expression levels of inflammatory factors in the fascia and muscle tissue after canine linea alba sutured. (A–C) mRNA expression levels of IL-1β, IL-6, and TNF-α in the fascia and muscle tissue at 1 day, 7 days, and 3 months post linea alba sutured. (D–F) Protein expression levels of IL-1β, IL-6, and TNF-α in the fascia and muscle tissue at 1 day, 7 days, and 3 months post linea alba sutured. [file Image_3.JPEG]
